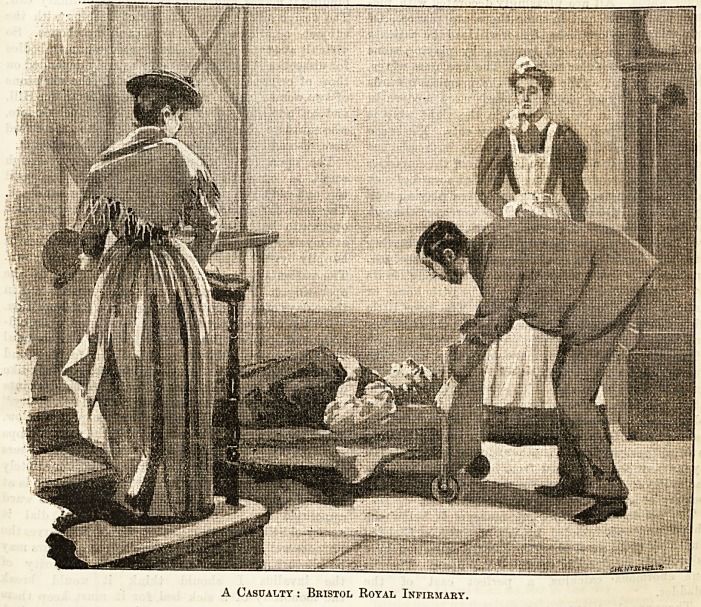# Royal Infirmary, Bristol

**Published:** 1894-03-03

**Authors:** 


### THE INSTITUTIONAL WORKSHOP.

### WITHIN THE HOSPITALS.

### THE ROYAL INFIRMARY, BRISTOL.

(BY A VAGRANT CORRESPONDENT.)

Now almost in the centre of but once an isolated building on the outskirts of the town stands the Royal Infirmary of Bristol City. It is an "away back" block, as our American cousins would say. Dates from the three-corned hat and wig and robber period of 1735. But for the two new wings at the back of the building, was roughly bound in many rags. Her husband propped her up and gradually helped her up the steps.

It was rather a pathetic sight, for the poor woman was evidently for the moment suffering more mental than physical trouble. The man was repeating, "It's all right, old gal; soon be out. I'll bring the kid to see you. Now, don't take on; it's all right." So the two hobbled on into the old hall. It was, indeed, an old hall, and opened on to a corridor which spread out on either side, as dark and dismal as the hall itself

A CASUALTY: BRISTOL ROYAL INFIRMARY.

is still Hogarthian enough to make one think of the architectural background to the "Rake's Progress" and other kindred works of that great painter.

I arrived at the principal entrance of the Infirmary in Marlborough Street just in time to see, minus the costume, an Hogarthian subject. A rickety coster type of a cart, dragged by a scraggy rat of a pony, straggled up to the doors. The driver was the husband of the obese lady seated in the back of the vehicle. The tailboard of the cart was slung on its chains and made a kind of spring seat for the lady, who was presently assisted down on to the steps of the hospital by the man. She was suffering from an injured leg, which

But for the cheering presence of a bright-faced neatly-clad nurse occasionally passing to and fro, this entrance to the Royal Infirmary would be depressing indeed. I was soon ushered into the Matron's room, and presented my letter of introduction. It was a relief to get into so bright and cheery a place from the gloom of the corridor. There was a brisk fire in the grate, charming etchings and photos on the walls, flowers and the latest magazines on the tables, and all the comfortable surroundings of a boudoir or drawing-room. I asked the Matron whether it would be more convenient for me to call later, but Miss Smith was quite ready to show me round the wards at once.

The latest improvement in the hospital is an excellent lift, which runs up to the top story by the central stairway. There are nine wards opening on to the corridor of the first and eight on the second floor. The third floor is devoted to nurses' rooms and the eye department of the hospital: this department is of recent date, and is under the control of Mr. F. R. Cross. This eminent Bristol oculist is honorary surgeon to the Bristol Eye Hospital.

On an average the wards have twelve beds in them. Though the rooms are small in comparison to later buildings of the kind, they are bright and cheerful, and seem much more comfortable and homely than larger wards. All are pleasingly decorated with pictures and plants, and in some there are pianos. Of an evening an accomplished Sister will lead the patients away from their physical troubles by playing some cheerful air or popular melody. The two new wings, which spread out at the back of the premises towards the garden or grass plot, have wards more modern in size, especially in height. One in the right wing is devoted to children, and has twenty cots. The scene which presented itself on my entrance impressed me considerably. It might have been one of the nurseries of the famous Brigham Young instead of a ward of sick children. Many of the young ones were playing with toys in their little beds, while others were sprawling on the floor building houses of wooden bricks. One hollow-eyed little fellow, propped up in a chair, was staring into the genial fire, building castles in the air. Another little creature lay pale and placid enough in its cot, and it seemed to me there would be little house or castle building for it in this world. The poor little thing was waiting patiently the call of the Great Master Builder.

The left wing of the infirmary was taken up by the museum and an Episcopalian chapel, a very prettily designed place of worship, with several artistic presentation windows. The centre window is excellent in colour, and is a memorial to one of the doctors who lost his life in the execution of his duties in attending to a diphtheric patient. Another window is dedicated to a young student who died from blood poisoning. On the walls of the chapel are several brass tablets to the memory of nurses who died at their post.

The wonderfully interesting museum is below the chapel, and was founded by Senior Surgeon Richard Smith as early as 1796. The calculi collection here is probably the finest in the three kingdoms. There is one enormous calculus, a perfect cast of the bladder.

There are also in this museum the most extraordinary bone specimens, the finest, I believe, next to the collection in the College of Surgeons. One specimen of ossifying sarcoma is probably the most unique extant. A very interesting though gloomy relic is that of the skeleton of one John Horwood, felon and murderer, who was hanged for the murder of his sweetheart. In our day his sentence would probably have been commuted to penal servitude. Horwood threw a stone at the head of his fiancée and then run away, and without seeing the damage he had done. The girl, who was seriously injured, was picked up and brought into the infirmary. At one time she seemed to be getting quite well, but she eventually, after several months in hospital, died. Horwood was arrested, tried, and condemned to be hanged. After the capital sentence follows, "and let his body be delivered to Mr. Richard Smith, of the City of Bristol, surgeon, to be dissected and anatomised." To show the stupid superstition of the time, "a number of foolish women with their children ascended the top of the scaffold after the culprit was turned off for the purpose of having their disease cured by touching the dead hands." These extracts from the *Bristol Mirror* of April 14th, 1821, and with all the surgeon's notes and drawings of the anatomising, are bound up in a book covered with the tanned skin of Horwood. Though gruesome, a very good durable binding it makes, and looks as tough as ordinary calf. The design on the cover is in strict keeping with the subject—a gallows in gold, skulls and crossbones. So difficult at that period was it to procure human bodies for dissection, that the infirmary entirely depended on those of criminals. A case containing the skeletons of two women who left a child to die on Brandon Hill, and an admirable print of Captain Samuel Godere, who murdered his brother in 1741, and was anatomised here, also add to the gloomy interest of the museum.

The operation theatre in the infirmary, though small, is excellent for its purpose. It has good top and side lights, and a gallery for the students. The room for placing the patient under anæsthetics opens into the theatre. By the lavatory recess in the wall are mysterious-looking iron rings, which are relics of happily bygone days when anæsthetics were unknown. The patients were then often chained down to the operating table, the only means the surgeons had in those days of keeping their subject in a fixed position.

All the rooms in the infirmary have large, open, old-fashioned grates. Singing kettles and the cheerful blaze of the fire make the wards look comfortable and homely. Noticeable in each ward is a large clock-like dial over the mantel and on the wall. A hand points to certain instructions printed on its face, "Casualty" &c., &c. The clock is worked by the porter below. When the hand moves a bell rings till the pointer stops —if, for instance, it stops at "casualty," the dressers and sisters in their respective wards, if not immediately occupied, hurry to look after the new comer, who is at once placed on the lift and ascended to the ward appointed for his or her reception. The dial is a very old-time invention, but is simple, and serves the purpose well. The ringing of the bell at all hours may be disturbing to some, but to the majority of the invalids I should think it would break the monotony of a sick bed, for it must keep them in touch with the outer working of the hospital. If that clock was in an American ward the patients would be laying wagers all day long, taking long odds on the casualties, short on the out-patients, or *vice versa*. I know that dial almost fascinated me as much as the many pretty intellectual-faced nurses flitting about the wards.

There is only one drawback to this admirably conducted institution, that is the want of proper accommodation for the nurses. These hard-worked little ladies have no common room where they can meet together in comfort and get away for a time from the atmosphere of the sick wards. There is a talk of building a large room on the roof. The sooner the better.

## Figures and Tables

**Figure f1:**